# Ear and Nose Abnormalities in Meningoencephalitis Associated With Relapsing Polychondritis: A Case Report

**DOI:** 10.7759/cureus.85254

**Published:** 2025-06-02

**Authors:** Tomoaki Taguchi, Soichiro Matsubara, Keiichi Nakahara, Hiroyuki Ohmori, Mitsuharu Ueda

**Affiliations:** 1 Neurology, Kumamoto University, Kumamoto, JPN; 2 Neurology, Yamaga Chuo Hospital, Yamaga, JPN

**Keywords:** case report, meningoencephalitis, pinna deformity, relapsing polychondritis, saddle nose

## Abstract

Relapsing polychondritis (RPC) is a rare autoimmune disease that affects cartilage and connective tissue, especially the ear and nose. The diagnosis of relapsing polychondritis is challenging due to its low incidence, and cases with meningoencephalitis as the predominant manifestation are particularly difficult to identify. We report a case of meningoencephalitis associated with relapsing polychondritis. A 67-year-old woman who had a 1.5-month history of fever, acute impaired consciousness, and nuchal rigidity was diagnosed with aseptic meningoencephalitis of uncertain etiology and referred to our hospital. Cerebrospinal fluid (CSF) analysis revealed pleocytosis and high levels of interleukin-6 (IL-6). Head magnetic resonance imaging (MRI) revealed a meningeal enhancement. Her ear and nose deformities were noted, and a biopsy from her pinna was conducted. Based on the histological findings, relapsing polychondritis was diagnosed with central nervous system (CNS) involvement. The symptoms were partially relieved by immunotherapy, and intrathecal interleukin-6 decreased. She has significantly improved and was discharged six months after the initiation of treatment. Since the most frequent clinical manifestation of relapsing polychondritis is auricular chondritis, this finding can become a clue to diagnosis. Neurologists, upon the diagnosis of meningoencephalitis of unknown cause, should carefully examine for nasal and, especially, ear abnormalities, considering the potential for central nervous system involvement in relapsing polychondritis.

## Introduction

Relapsing polychondritis (RPC) is a rare immune-mediated systemic disease. This disease is characterized by recurrent flares of cartilaginous and proteoglycan-rich tissues, including the ear, the nose, peripheral joints, fibrocartilage at axial sites, and the cartilage of the tracheobronchial tree. These flares result in progressive anatomical deformation and the functional impairment of the involved structures [1]. In particular, the involvement of the heart and tracheobronchial tree can sometimes lead to sudden death. Some symptoms outside the cartilage, such as headache, dementia, stroke, and parkinsonism, are thought to be derived from nervous system involvement, and these symptoms occur in only 3% of RPC patients [2]. One such manifestation is aseptic meningitis, characterized by cerebrospinal fluid (CSF) pleocytosis. The diagnosis of RPC has been challenging due to its low incidence, particularly when meningoencephalitis is the predominant clinical presentation. We herein report a patient with RPC that was diagnosed following the onset of meningoencephalitis. The recognition of characteristic ear and nasal deformities can be crucial for early diagnosis, especially in atypical presentations with predominant neurological symptoms, such as the case we describe below.

## Case presentation

**Figure 1:**
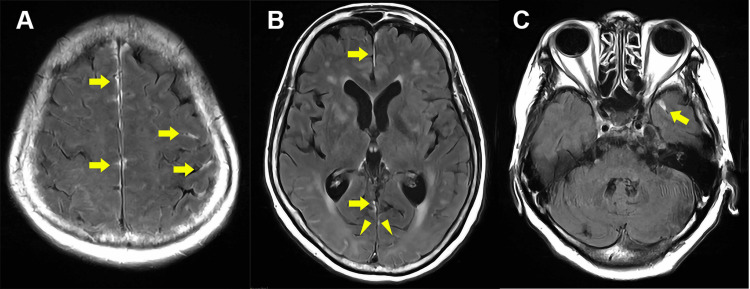
Contrast-enhanced head MRI Contrast-enhanced fluid-attenuated inversion recovery (FLAIR) axial images (A-C) demonstrate abnormal enhancement along the gyri of the interhemispheric frontal and parietal lobes, as well as within the sulci of the frontal and temporal lobes (arrows) and high T2 signal intensity without contrast enhancement in the cerebral white matter, predominantly in the occipital lobe (arrowheads) MRI: magnetic resonance imaging

A 67-year-old woman presented with a 1.5-month history of acute-onset fever, difficulty walking, and impaired consciousness. She initially consulted a local physician who tested her for SARS-CoV-2 polymerase chain reaction (PCR), which was negative. Despite this, her symptoms progressively worsened. She was subsequently admitted to another hospital. As she had a high fever and confusional state with no infection features in her lung and urinary tract on admission, they administered antibiotics for presumed meningoencephalitis. Due to a lack of clinical improvement, she was referred to our hospital for re-evaluation and treatment. She had no family history related to the present illness. On admission, the patient’s body temperature was 37.8℃, and the other vital signs were stable. She had no hoarseness, wheezes, swollen joints, or heart murmur. The neurological examination identified impaired consciousness (Glasgow Coma Scale: 13 E4V4M5), startle response, emotional lability, confabulation, and meningeal irritation. Cranial nerve examination revealed no abnormal findings. There was no involuntary movement, but she could not follow specific movement instructions. Deep tendon reflexes were also normal. As shown in Table 1, blood tests showed high inflammatory markers, including leukocytosis (12,600/μL) and elevated C-reactive protein (5.94 mg/dL), as well as anemia, with normal renal and cardiac functions. Cerebrospinal fluid (CSF) analysis revealed pleocytosis (23 mononuclear cells/μL and two polymorphonuclear cells/μL) and positive oligoclonal bands, and the levels of interleukin-6 (IL-6) were elevated to a maximum of 601 pg/mL. There were no significant findings in the culture of various viral antibody titers, bacteria, fungi, or tuberculosis, and atypical cells were absent on a cytodiagnosis. Electroencephalography showed a poorly organized posterior dominant rhythm of 8-9 Hz and no epileptiform abnormality. Head magnetic resonance imaging (MRI) showed patchy abnormal enhancement along the gyri and noncontrast high T2 signals in the cerebral white matter with occipital lobe predominance (Figure 1A-1C). In brain perfusion single-photon emission computed tomography (SPECT), relatively high blood flow in the medial side of the temporal lobe was found.

**Table 1:** Laboratory findings and diagnostic examination BNP, brain natriuretic peptide; sIL-2R, soluble interleukin-2 receptor; IgG, immunoglobulin G; IgM, immunoglobulin M; IgA, immunoglobulin A; ANA, antinuclear antibody; Anti-dsDNA Ab, anti-double-stranded DNA antibody; Anti-SSA Ab, anti-Sjögren’s syndrome-related antigen A antibody; MPO/PR3-ANCA, myeloperoxidase/proteinase 3-antineutrophil cytoplasmic antibody; Anti-TPO Ab, anti-thyroid peroxidase antibody; HBsAg, hepatitis B surface antigen; Anti-HCV Ab, anti-hepatitis C virus antibody; HIV Ab, human immunodeficiency virus antibody; HSV, herpes simplex virus; VZV, varicella zoster virus; TB, tuberculosis; CT, computed tomography; MRI, magnetic resonance imaging; 99mTc-ECD SPECT, technetium 99m-ethyl cysteinate dimer single-photon emission computed tomography

Examination	Results	Reference value
Peripheral blood		
Hemoglobin	9.3 g/dL	11.6-14.8 g/dL
Platelet	43.6×10^4^/μL	15.8-34.8×10^4^/μL
White blood cells	12.6×10^3^/μL	3.3-8.6×10^3^/μL
Blood chemistry		
Total protein	7.3 g/dL	6.6-8.1 g/dL
Albumin	2.7 g/dL	4.1-5.1 g/dL
Total bilirubin	0.5 mg/dL	0.4-1.5 mg/dL
Aspartate aminotransferase	23 U/L	13-30 U/L
Alanine aminotransferase	22 U/L	7-23 U/L
Lactate dehydrogenase	211 U/L	124-222 U/L
Blood urea nitrogen	21.2 mg/dL	8-20 mg/dL
Cr	0.85 mg/dL	0.46-0.79 mg/dL
Na	138 mEq/L	138-145 mEq/L
K	4.3 mEq/L	3.6-4.8 mEq/L
Cl	104 mEq/L	101-108 mEq/L
Vitamin B1	29 ng/mL	24-66 ng/mL
BNP	6.5 pg/mL	<18.4 pg/mL
Blood glucose	143 mg/dL	73-109 mg/dL
Immunoserological tests		
C-reactive protein	5.94 mg/dL	<0.14 mg/dL
sIL-2R	464 U/mL	121-613 U/mL
IgG	1,916 mg/dL	861-1,747 mg/dL
IgM	393 mg/dL	50-269 mg/dL
IgA	68 mg/dL	93-393 mg/dL
Interleukin-6	75.8 pg/mL	≤7 pg/mL
Rheumatoid factor	Negative	
ANA	3.1	<10
Anti-dsDNA Ab	2.2 IU/mL	<12 IU/mL
Anti-SSA Ab	Negative	
MPO/PR3-ANCA	Negative	
Anti-TPO Ab	Negative	
Microbiological test		
HBsAg/anti-HCV Ab/HIV Ab	Negative	
β-d-glucan	Negative	
Tuberculosis-specific interferon-*γ*	Negative	
Blood culture	Negative	
Cerebrospinal fluid (CSF)		
Cell count (percentage of mononuclear cells)	25/μL (92%)	
Protein	60.8 mg/dL	8-43 mg/dL
Glucose	62 mg/dL	50-75 mg/dL
IgG index	0.67	
Interleukin-6	49.2 pg/mL	≤7 pg/mL
Oligoclonal band	Positive	
CSF culture	Negative	
HSV/VZV/TB PCR	Negative	
Cytological examination	Negative	
Echocardiogram	Normal	
Ultrasonography of arthra	Normal	
Contrast-enhanced whole-body CT	Normal	
Upper gastrointestinal endoscopy	Normal	
Nerve conduction study	Normal	
Electroencephalogram	Only a slight abnormality	
Head contrast-enhanced MRI	Abnormal	
Spine contrast-enhanced MRI	Normal	
99mTc-ECD SPECT	Only a slight abnormality	

**Figure 2:**
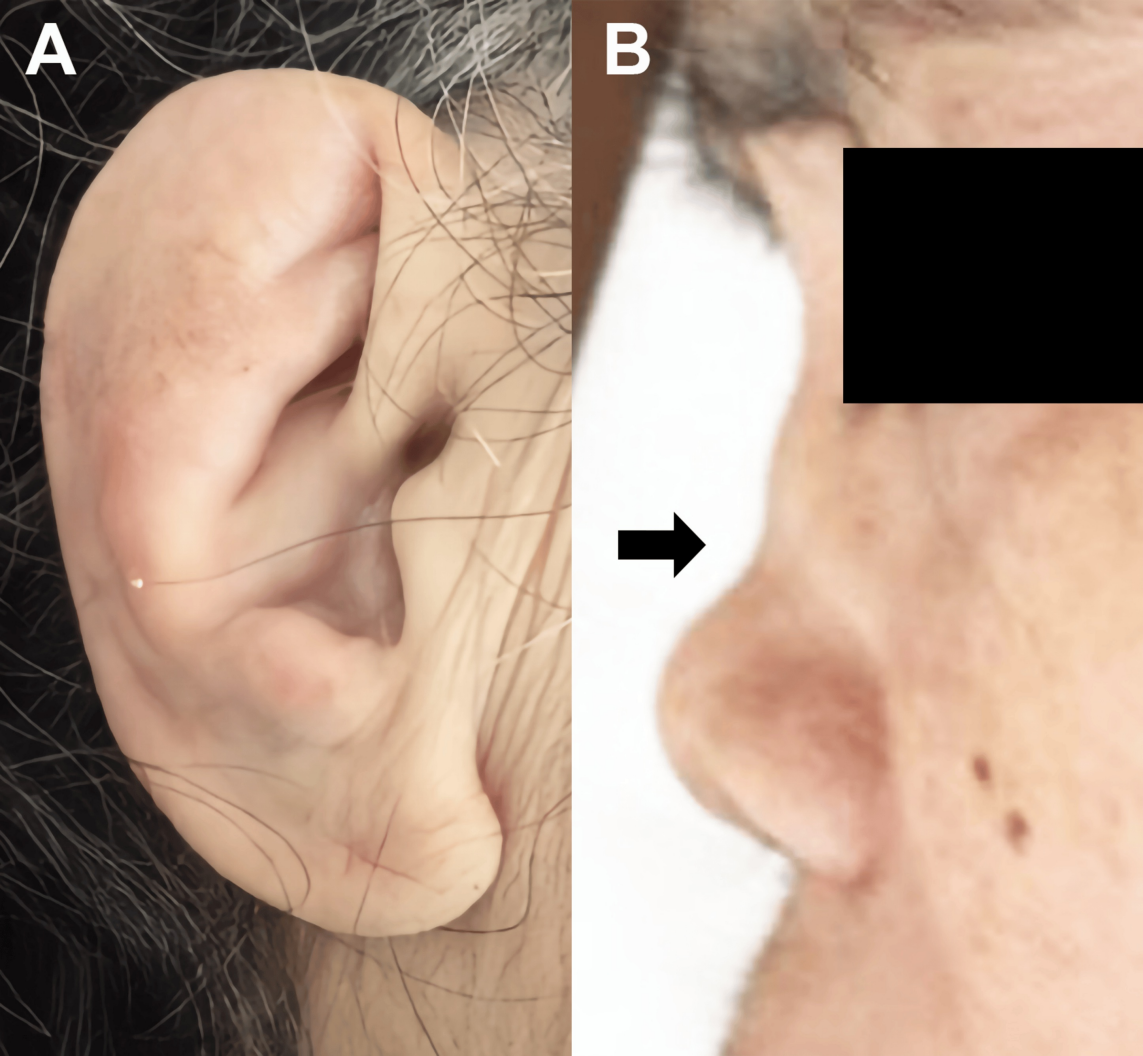
Ear and nose deformities The swelling and deformation of both ears are observed (A), and the nose exhibits a saddle deformity (B)

**Figure 3:**
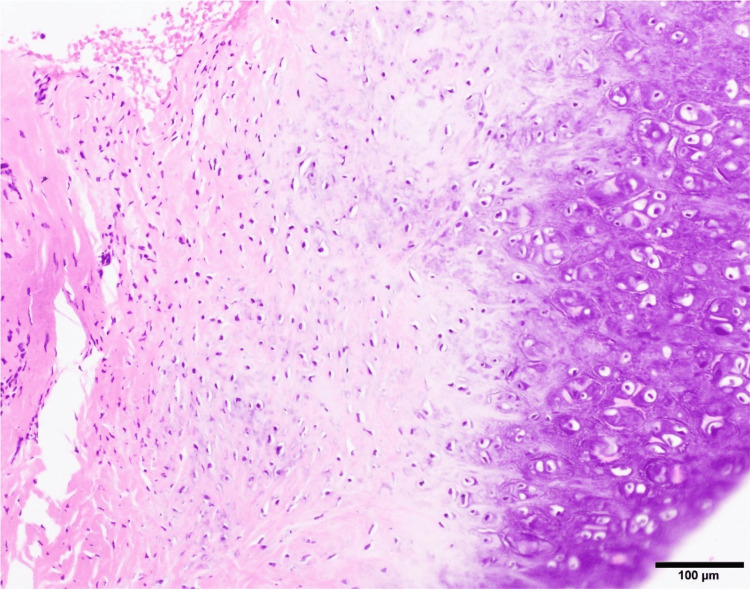
A pathological image from the right auricular cartilage Hematoxylin and eosin (H&E) staining of a biopsy specimen from the right auricular cartilage. The infiltration of chronic inflammatory cells into the perichondrial tissues is observed, along with decreased basophilic staining in the cartilage matrix (scale bar: 100 μm)

Despite empirical treatment for presumed viral or fungal meningoencephalitis, the patient showed no clinical improvement. Upon reassessment, we noted that she had ear deformities with erythema (Figure 2A), saddle nose (Figure 2B), and scleritis. Further review of the patient’s history revealed the presence of recurrent scleritis for the past two years. Despite this ongoing inflammatory condition, the ear and nose abnormalities had developed insidiously, as neither the patient nor her husband had noticed them before the current admission. A pinna biopsy revealed degenerative changes with lymphoid infiltration (Figure 3).

**Figure 4:**
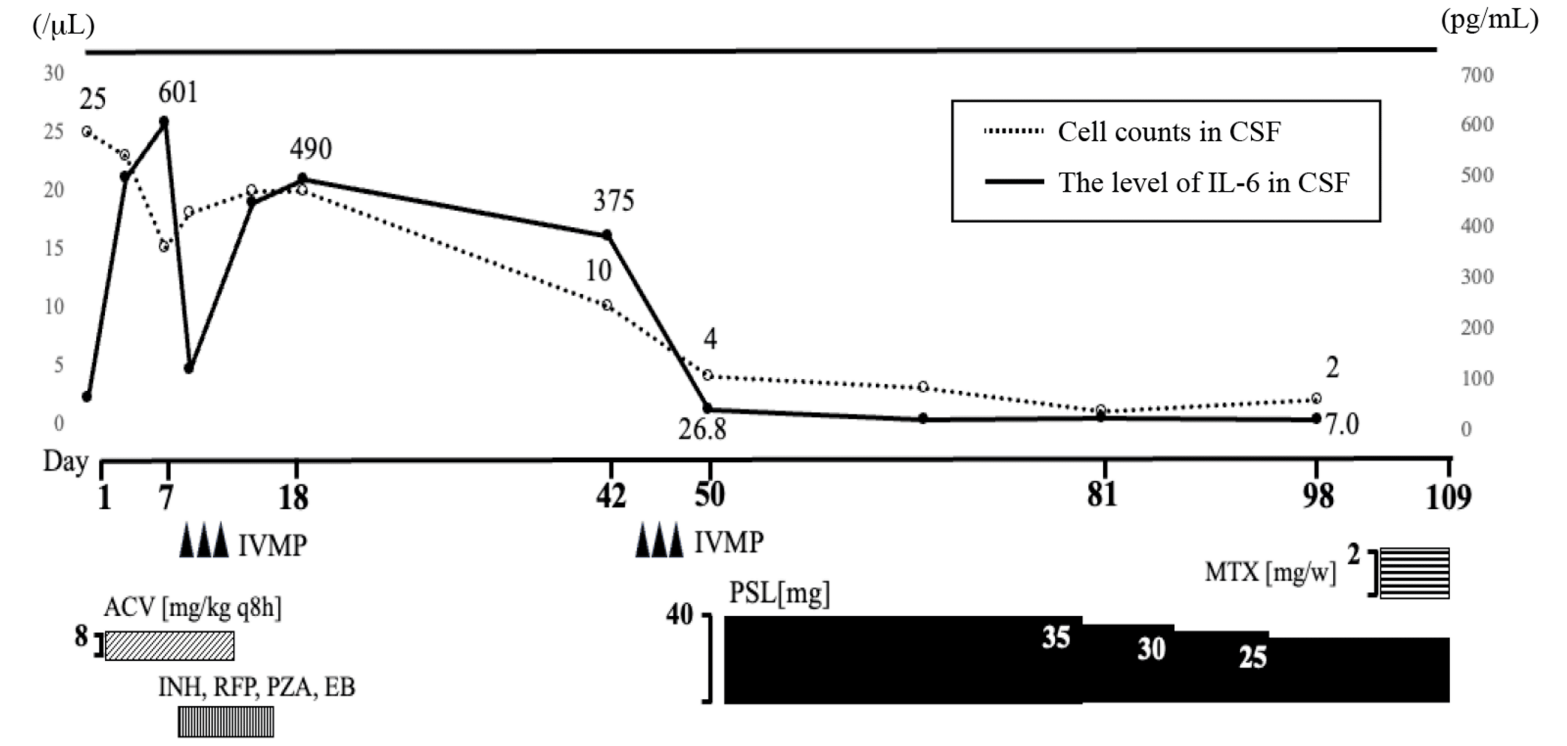
Therapeutic interventions and changes in cerebrospinal fluid cell counts and interleukin-6 levels during hospitalization The graph shows the relationship between therapeutic interventions and changes in cerebrospinal fluid (CSF) cell counts (dotted line) and interleukin-6 (IL-6) levels (solid line). Note the marked decrease in both parameters following steroid pulse therapy and subsequent immunosuppressive treatment IVMP, intravenous methylprednisolone; ACV, acyclovir; INH, isoniazid; RFP, rifampicin; PZA, pyrazinamide; EB, ethambutol; PSL, prednisolone; MTX, methotrexate

The principal symptoms and findings of the case can be summarized as follows: subacute-onset impaired consciousness, systemic inflammatory response without infection features, CSF pleocytosis, high protein level, elevated IL-6, patchy abnormal enhancement in the meninges and noncontrast high T2 signals in the cerebral white matter with occipital lobe predominance, ear and nose deformities with inflammation, and scleritis. She was thus diagnosed with RPC according to previously reported diagnostic criteria [3]. After treatment with high-dose prednisolone and methotrexate (MTX), her symptoms, such as fever, scleritis, and swollen ears, improved without saddle nose. While temporal disorientation remained, symptoms of emotional lability and confabulation resolved. As shown in Figure 4, the IL-6 levels in CSF decreased to 7.0 pg/mL after treatment. She was then transferred to another hospital, and the prednisolone dose was gradually reduced. She has significantly improved and was discharged six months after the initiation of treatment.

## Discussion

RPC is a rare immune-mediated systemic disease characterized by inflammation in cartilaginous tissues and other major organs [1]. While RPC may impact multiple organ systems, central nervous system (CNS) manifestations are uncommon. The estimated incidence of RPC is 3.5 per 1,000,000 people per year, with RPC involving the central nervous system (CNS) being even rarer [4]. While a prompt diagnosis of meningoencephalitis associated with RPC is challenging because of its rarity, early diagnosis and prompt therapeutic intervention are essential to prevent not only irreversible brain damage but also other life-threatening conditions associated with RPC, such as severe bronchial stenosis or intractable cardiac failure because of valve regurgitation. Since this patient presented with subacute fever and impaired consciousness, we needed to consider various differential diagnoses, including encephalitis or meningoencephalitis due to pathogenic microorganisms (particularly tuberculosis, fungi, and the herpes viruses), CNS vasculitis, and malignancy. The patient had no history of neoplasm, recent vaccination, or drug use. Additionally, no findings suggestive of an infectious etiology or malignancy were observed in various culture tests, nucleic acid tests, cerebrospinal fluid cytology, and imaging examinations. Despite empirical treatment for presumed viral or fungal meningoencephalitis, the patient showed no clinical improvement. Upon reassessment, we noted two significant physical findings: saddle nose deformity and auricular inflammation, characterized by swelling and erythema. Based on the histological examination of auricular tissue, we ultimately diagnosed RPC. Although definitive diagnostic criteria for RPC-related meningoencephalitis do not exist, previous case reports indicated that diagnosis typically relies on excluding alternative causes and observing treatment response [5,6]. This diagnostic approach was consistent with our case. In the previous report, RPC patients with CNS involvement mainly presented with meningoencephalitis or meningitis [7]. In some cases, cranial nerve involvement is also observed. Since auricular chondritis is the most frequent clinical manifestation of RPC, present in 83%-95% of cases during the course of the disease, it represents an important diagnostic clue [8,9]. Furthermore, studies have shown that auricular involvement is significantly more common in RPC patients with CNS manifestations compared to those without CNS involvement, making ear examination particularly valuable for identifying patients at risk for neurological complications [7].

Little is known about the mechanism of CNS complications in RPC, although it is thought that cartilage-specific autoimmunity may play a crucial role in its pathogenesis. It is speculated that vasculitis in RPC occurs due to autoimmune mechanisms against proteoglycan, and it is not unusual for vasculitis to occur throughout the body [10]. Collagen fibrils are also known to be present in the arachnoid, and it is also speculated that inflammation may occur there in RPC [11]. Although anti-neutral glycolipid antibody was negative in our case, recent reports have shown GluRε2 and neutral glycolipid antibodies being positive in serum and CSF in RPC patients with limbic encephalitis [12,13]. These autoantibodies are produced in response to antigen exposure associated with neuronal damage, leading to CNS lesions [14]. Considering these cases, these various autoantibodies may play a role in CNS complications.

There are no established optional therapeutic approaches to RPC with meningoencephalitis. Usually, steroid therapy, including steroid pulse therapy, is administered. Since ineffectiveness or flare-ups may occur with steroid monotherapy, the combination of immunosuppressive agents such as azathioprine, MTX, or cyclophosphamide is needed during the course of steroid therapy [15]. In this case, combination therapy with steroids and MTX caused a good clinical course without any recurrence.

IL-6 is a cytokine involved in B-cell differentiation, antibody production, and the T-helper 17 (TH17) pathway of T-cell development [16,17]. Some reports have suggested that higher intrathecal IL-6 levels in autoimmune encephalitis are associated with unfavorable prognosis and high disease activity [18]. However, other studies propose that elevated IL-6 levels may directly contribute to nervous system damage [19]. In addition, Kawai et al. have reported beneficial effects of the anti-IL-6 receptor antibody tocilizumab in two patients with refractory RPC [20]. Considering these reports, intrathecal IL-6 levels may serve as a useful disease activity marker for RPC with CNS involvement. Unfortunately, in Japan, anti-IL-6 receptor antibody medications such as tocilizumab cannot be used in routine clinical practice covered by health insurance, obliging us to resort to combination therapy with prednisolone and MTX. Nevertheless, intrathecal IL-6 levels decreased after treatment, correlating with symptom improvement, similar to previous reports [5,6]. Notably, despite the initially elevated IL-6 levels, our patient had a favorable clinical course, possibly due to early diagnosis.

## Conclusions

In this case, we made a relatively early diagnosis of meningoencephalitis associated with RPC upon noticing the swelling and deformity of the pinna and nose, leading to a favorable clinical course despite elevated IL-6 levels. We believe that pinna and nose chondritis can serve as important clues for early diagnosis, given their frequent manifestation in RPC. Needless to say, the early diagnosis of RPC is important to prevent irreversible damage. Neurologists, upon the diagnosis of meningoencephalitis of unknown cause, should carefully examine for nasal and, especially, ear abnormalities, considering the potential for CNS involvement in RPC.
